# Cardiovascular Outcome in Patients with Major Depression: Role of Obstructive Sleep Apnea Syndrome, Insomnia Disorder, and COMISA

**DOI:** 10.3390/life14050644

**Published:** 2024-05-19

**Authors:** Matthieu Hein, Benjamin Wacquier, Matteo Conenna, Jean-Pol Lanquart, Camille Point

**Affiliations:** 1 Hôpital Universitaire de Bruxelles, Service de Psychiatrie et Laboratoire du Sommeil, Université Libre de Bruxelles, ULB, 1070 Brussels, Belgium; benjamin.wacquier@hubruxelles.be (B.W.); matteo.conenna@hubruxelles.be (M.C.); secmed.psy.erasme@hubruxelles.be (J.-P.L.); camille.point@hubruxelles.be (C.P.); 2 Laboratoire de Psychologie Médicale et Addictologie (ULB312), Université Libre de Bruxelles, ULB, 1020 Brussels, Belgium; 3 Route de Lennik, 1070 Anderlecht, Belgium

**Keywords:** cardiovascular risk, COMISA, insomnia disorder, obstructive sleep apnea syndrome, major depressive disease

## Abstract

In this study, the 10-year cardiovascular risk associated with comorbid sleep disorders (insomnia disorder, obstructive sleep apnea syndrome, and COMISA [comorbid insomnia and sleep apnea]) was investigated for patients with major depression. To enable our analysis, 607 patients with major depression were selected from the data register of the Sleep Unit. High 10-year cardiovascular risk was considered present when the Framingham Risk Score was ≥10%. The 10-year cardiovascular risk associated with comorbid sleep disorders has been assessed using logistic regression analyzes. High 10-year cardiovascular risk is significant (40.4%) in patients with major depression. After successive introduction of the different confounders, multivariate logistic regressions showed that for patients with major depression high 10-year cardiovascular risk was significantly associated with COMISA but was not significantly associated with insomnia disorder or obstructive sleep apnea syndrome alone. Thus, these results highlight the existence of a negative synergistic action between insomnia disorder and obstructive sleep apnea syndrome on the 10-year cardiovascular risk in patients with major depression, which demonstrates the importance of researching and treating COMISA to improve the prognosis of this specific population subgroup characterized by higher cardiovascular morbidity and mortality.

## 1. Introduction

The available studies seem to indicate the existence of a special relationship between major depressive disease (MDD) and cardiovascular diseases [1]. Indeed, the occurrence of major depressive episodes is frequent in individuals with cardiovascular diseases whereas the rate of cardiovascular diseases is significant for MDD patients [2,3]. In addition, the presence of comorbid cardiovascular diseases is associated with higher severity of major depressive episodes and the severity of cardiovascular diseases is greater in cases of comorbid MDD [4,5]. Moreover, in the literature, there seems to be evidence in favor of a negative impact of MDD on the cardiovascular outcome for both non-cardiovascular and cardiovascular patients [6,7]. However, despite the existence of this increased cardiovascular risk associated with MDD, the currently available studies seem to indicate that psychotherapeutic or antidepressant treatments are only associated with a partial improvement in cardiovascular prognosis for MDD patients [8,9]. In this context, the identification of potential cofactors involved in this increased cardiovascular risk associated with MDD seems to be essential to open new perspectives for the prevention of negative cardiovascular consequences related to this psychiatric disorder.

Obstructive sleep apnea syndrome (OSAS) and insomnia disorder (ID) are cardiovascular risk factors demonstrated for the general population [10,11]. Furthermore, following the existence of data supporting a potential negative synergistic action of these two sleep disorders, comorbid insomnia and *sleep* apnea (COMISA) appear to be associated with a more severe cardiovascular risk than OSAS or ID alone [12,13]. However, although the rate of OSAS and ID is significant for MDD patients [14,15], only a limited number of studies have currently investigated the risk of cardiovascular diseases associated with these sleep disorders for this specific population subgroup [16,17]. Furthermore, despite the existence of a potential negative synergy between OSAS and ID [18], the impact of COMISA on cardiovascular risk for MDD patients has not been studied yet. Thus, based on these elements, additional studies appear to be necessary to provide a better understanding of the potential role played by OSAS, ID, and COMISA in increased cardiovascular risk for MDD patients.

Our main objective for this study was to explore the 10-year cardiovascular risk associated with OSAS, ID, and COMISA for MDD patients. Given previous studies available in the literature for other subgroups of patients [12,13,18,19], the hypothesis of this study was that, unlike OSAS or ID alone, only COMISA is associated with an increased 10-year cardiovascular risk for MDD patients. Obtaining reliable data on the 10-year cardiovascular risk associated with comorbid sleep disorders (OSAS, ID, and COMISA) for MDD patients was the aim of this approach to enable better management by health professionals of this specific population subgroup characterized by higher cardiovascular morbidity and mortality.

## 2. Material and Method

### 2.1. Recruitment of MDD Patients

After applying the extraction criteria from Table 1 [20,21], 607 MDD patients referred for polysomnography between 1 January 2002 and 31 December 2020 were selected from the data register of the Sleep Unit of the University Hospital of Brussels. We focused only on MDD patients in whom the occurrence of comorbid sleep disorders appeared to be associated with increased cardiovascular risk [16,17]. Before being hospitalized in the Sleep Unit, these MDD patients underwent a prior ambulatory assessment by a physician specialized in sleep medicine, as described in the Supplementary Material—S1.

### 2.2. Clinical Assessment of MDD Patients

A comprehensive medical check-up (medical history combined with physical examination, medical record review, and complementary tests) was carried out in all these MDD patients during their hospitalization in the Sleep Unit to systematically identify their potential traditional cardiovascular risk factors and their other comorbid medical pathologies (Supplementary Material—S2) [22,23]. Following this somatic check-up, the Framingham Risk Score (FRS) was measured in all these MDD patients to determine the 10-year cardiovascular risk: FRS < 10% for low 10-year cardiovascular risk and FRS ≥10% for high 10-year cardiovascular risk [24]. The FRS was chosen to calculate the 10-year cardiovascular risk in this study because this 10-year risk score of developing clinical cardiovascular diseases is regularly applied in the subgroup of MDD patients [25,26].

Subsequently, a physician specialized in psychiatry assigned to the Sleep Unit conducted a systematic psychiatric examination in all these MDD patients to confirm the diagnosis of major depressive episodes and screen for potential other psychiatric disorders, according to the diagnostic criteria of *Diagnostic and Statistical Manual of Mental Disorders IV-TR* (before 2013) or *Diagnostic and Statistical Manual of Mental Disorders 5* (after 2013) [20,21]. Finally, the subjective complaints of depression, insomnia, and daytime sleepiness of all these MDD patients were assessed using a standardized series of questionnaires specific to our Sleep Unit (Supplementary Material—S3) [27,28,29].

### 2.3. Sleep Assessment of MDD Patients

In all these MDD patients, a systematic investigation of sleep habits and sleep-related complaints was carried out during a standardized interview by a physician specialized in sleep medicine in order to research for the presence of signs suggestive of the main sleep pathologies.

During their hospitalization in the Sleep Unit, all these MDD patients underwent polysomnography, meeting international recommendations regarding the installation and use of recording equipment (Supplementary Material—S4) [30]. Afterwards, visual scoring of all these polysomnographic recordings was carried out by specialized technicians according to international scoring recommendations. (Supplementary Material—S5) [31,32,33].

Finally, thanks to this complete sleep assessment, all potential comorbid sleep disorders were systematically diagnosed in MDD patients selected for this study: ID (Supplementary Material—S6), OSAS (apnea-hypopnea index ≥ 5/h), COMISA (ID + OSAS), moderate to severe periodic limb movement syndrome (periodic limb movement index ≥ 15/h), restless legs syndrome, and short sleep duration (<6 h) [34,35,36,37,38].

### 2.4. Statistical Analyzes

To enable statistical analyzes using Stata 14 software, the sample of MDD patients was divided between a subsample with low 10-year cardiovascular risk (FRS < 10%) and a subsample with high 10-year cardiovascular risk (FRS ≥ 10%) [24].

Given the asymmetric distribution of the majority of continuous data in this study, medians (P25–P75) were used for their description and Wilcoxon tests were used for group comparisons. For categorical data, percentages (%) were used for their description and Chi square tests were used for group comparisons.

Univariate logistic regressions were used to determine the 10-year cardiovascular risk associated with comorbid sleep disorders (categorized: No ID and/or OSAS, ID alone, OSAS alone, COMISA) and the potential confounders (Supplementary Material—S7). Concerning multivariate logistic regressions, potential adjustments of the 10-year cardiovascular risk associated with comorbid sleep disorders were carried out following successive introduction of significant confounders during the univariate analyzes.

For the final model, the adequacy and the specificity were verified, respectively, using the Hosmer–Lemeshow test and the Link test.

A *p*-value < 0.05 was used to identify significant results.

## 3. Results

### 3.1. Polysomnographic Parameters

The stage 1%, the wake after sleep onset %, the micro-arousal index, the apnea–hypopnea index, the oxygen desaturation index, and the total time under 90% of SaO_2_ were higher for MDD patients with high 10-year cardiovascular risk (Table 2). On the other hand, the sleep efficiency, the sleep period time, the total sleep time, the stage 3%, and the REM % were lower for MDD patients with high 10-year cardiovascular risk (Table 2). Finally, other polysomnographic parameters did not differ significantly between the two groups of MDD patients.

### 3.2. Demographic Parameters

The 10-year cardiovascular risk was high in 40.4% (n = 245) of MDD patients from our sample (Table 3). In addition, this high 10-year cardiovascular risk for MDD patients was significantly associated with male sex, overweight, obesity, age ≥60 years, tobacco consumption, drinking alcohol, conventional cardiovascular risk factors, aspirin use, CRP levels ≥1 mg/L, restless legs syndrome alone or combined with periodic limb movement index ≥15/h, OSAS alone, and COMISA (Table 3). Furthermore, compared to those with high 10-year cardiovascular risk, MDD patients with low 10-year cardiovascular risk presented lower age/CRP levels/body mass index and higher Insomnia Severity Index/Beck Depression Inventory scores (Table 3). Other demographic parameters did not differ significantly between the two groups of MDD patients (Table 3). Finally, the prevalence of ID alone, OSAS alone, and COMISA was, respectively, 42.5%, 17.5%, and 24.5% in our population of MDD patients (Table 3).

### 3.3. Multivariate Regression Analyzes

After adjustment following successive introduction of the previously identified confounders, multivariate logistic regressions demonstrated that for MDD patients high 10-year cardiovascular risk was significantly associated with COMISA but was not significantly associated with ID or OSAS alone (Table 4).

## 4. Discussion

Based on the results of this study, we have shown that MDD patients are a specific population subgroup characterized by a potential unfavorable cardiovascular outcome. Indeed, a high 10-year cardiovascular risk was present in 40.4% of MDD patients from our sample, which is superior to the rate observed in the general population [39]. However, this rate of MDD patients with high 10-year cardiovascular risk is superior to the rate of the studies by Yakar et al. (2018) and Garcia-Portilla et al. (2009), which could be due to the recruitment of patients with mood disorders at lower cardiovascular risk in these two studies [40,41]. Indeed, the patients with mood disorders recruited in these two studies had a younger age, less severe cardiometabolic comorbidities, and a healthier lifestyle than those from our study [40,41]. Moreover, this rate of MDD patients with high 10-year cardiovascular risk is inferior to the rate of the study by Slomka et al. (2012), which could be due to the recruitment of patients with mood disorders characterized by a more severe cardiovascular profile in this study [42]. Indeed, the patients with mood disorders recruited in this study presented an older age, more severe cardiometabolic comorbidities and more unhealthy lifestyle habits than those from our study [42]. However, despite these potential differences depending on the recruited population of MDD patients, the presence of this high cardiovascular risk for this specific population subgroup seems to be induced by the activation of several pathophysiological mechanisms deleterious for the cardiovascular system (dysregulation of the hypothalamic–pituitary–adrenal axis, activation of inflammatory mechanism, genetic factors, autonomic nervous dysfunction, alteration of serotonergic systems, microRNA alterations, Omega-3 disorder, and change in gut microbiota) [43]. Thus, given the existence of this high 10-year cardiovascular risk for MDD patients, a systematic identification of traditional and specific cardiovascular risk factors is essential in this specific population subgroup.

Consistent with the data currently available in the literature, we confirmed that ID (67.0%) and OSAS (42.0%) are frequent comorbidities for MDD patients [44,45]. Furthermore, in this study, we demonstrated that 58.4% of MDD patients with OSAS presented a comorbid ID, which seems to indicate a more frequent occurrence of insomnia complaints in cases of OSAS for MDD patients than for the other subpopulations currently studied for COMISA [12,13,18]. Furthermore, similar to other available studies [12,13,18], we demonstrated that high 10-year cardiovascular risk was significantly associated with COMISA but was not significantly associated with ID or OSAS alone. However, pathophysiologically, this high prevalence of COMISA and its potential role in unfavorable cardiovascular prognosis for MDD patients may be explained by different elements. First, some pathophysiological mechanisms specific to MDD could help to better understand this frequent co-occurrence of ID and OSAS for this specific subgroup of patients [46,47,48,49]. Indeed, for MDD patients, the existence of a vulnerability to stress-related disturbances and some neurobiological factors (alterations in monoaminergic neurotransmission, genetic predisposition, dysregulation of the hypothalamic–pituitary–adrenal axis, and hyperarousal state) have a central role in this frequent occurrence of ID whereas changes in upper airway tone secondary to alterations in serotonergic neurotransmission and potential weight gain secondary to psychotropic treatments are associated with the development of repeated obstructive respiratory events favoring the occurrence of OSAS [46,47,48,49]. Secondly, the demonstration of this high 10-year cardiovascular risk limited only to COMISA in this study seems to indicate the existence of a negative synergistic action between these two sleep disorders that could be one of the mechanisms involved in the development of cardiovascular diseases for MDD patients. However, based on the available data [50,51,52], the occurrence of a cumulative effect of pathophysiological mechanisms deleterious for the cardiovascular system present in both ID and OSAS (activation of inflammatory pathways, hyperactivation of the sympathetic nervous system, and dysregulation of the *neuroendocrine system*) appears to be the most probable explanation for the existence of this negative synergistic action of COMISA on the 10-year cardiovascular risk demonstrated for our sample of MDD patients. Thus, for MDD patients, systematic screening and targeted treatment of COMISA appear to need to be implemented to improve the prognosis of this specific population subgroup characterized by higher cardiovascular morbidity and mortality.

Therapeutically, the highlighting of this high 10-year cardiovascular risk associated with COMISA could open new therapeutic perspectives to improve the cardiovascular prognosis of MDD patients. Indeed, although there are elements in favor of a possible beneficial action of psychotherapeutic and pharmacological treatments of MDD on cardiovascular prognosis [53,54], it is essential to adequately manage conventional and specific cardiovascular risk factors for MDD patients [55]. In this context, for MDD patients with COMISA, it seems necessary to establish a combined treatment of ID and OSAS to avoid the persistence of pathophysiological mechanisms deleterious for the cardiovascular system following incomplete treatment of COMISA [50,51,52]. Among the therapeutic options available for COMISA, the combination of cognitive behavioral therapy for insomnia and continuous positive airway pressure therapy associated with lifestyle modifications currently remains the recommended first-line treatment [56,57]. Indeed, pharmacological treatments for insomnia are only recommended as a second line due to their potential side effects and their potential use limitations, whereas alternative treatments to continuous positive airway pressure therapy have few studied in the management of COMISA [58,59,60]. However, in the case of initiation of this combined treatment for MDD patients with COMISA, it will be necessary to anticipate the possible negative effect of MDD on compliance with cognitive behavioral therapy for insomnia and continuous positive airway pressure therapy to minimize the risk of treatment failure in this specific population subgroup [61,62]. Furthermore, alongside the potential positive impact of COMISA treatment on cardiovascular prognosis, it has been shown that for MDD patients, treatments for ID and OSAS may reduce the severity of depressive symptoms, which justifies treating these sleep disorders even if they occur alone [63,64]. Finally, based on these different arguments, it is, therefore, necessary to consider the specificities of MDD patients during the initiation of combined treatment of COMISA to avoid potential compliance problems that could negatively impact both cardiovascular and clinical outcomes in this specific population subgroup.

A summary of the comparison of the main results of this study with the literature is available in Table 5.

### Limitations

Given the lack of direct verification of data collected retrospectively from MDD patients selected for this study, the results obtained in this study need to be confirmed by future prospective studies. Additionally, since we did not extract patients with psychiatric disorders other than MDD, the results of this study may only be applied to MDD patients. Furthermore, although there is evidence for using the FRS to assess the 10-year cardiovascular risk for MDD patients [25,26,40,41,42], this risk score was initially developed to investigate cardiovascular outcome in the general population, which justifies taking into account its potential use limitations for this particular subpopulation when interpreting our results [65]. Finally, the data register of the Sleep Unit of the University Hospital of Brussels only contains MDD patients who were referred for polysomnography, which may prevent extrapolation of the findings of this study to all MDD patients.

## 5. Conclusions

A FRS ≥10% was present in 40.4% of MDD patients from our sample, which confirms that this specific population subgroup is at high 10-year cardiovascular risk. Furthermore, in our sample of MDD patients, we have shown that high 10-year cardiovascular risk was significantly associated with COMISA but was not significantly associated with ID or OSAS alone, which highlights the importance of researching and treating COMISA to improve the prognosis of this specific population subgroup characterized by higher cardiovascular morbidity and mortality.

## Figures and Tables

**Table 1:** Selection criteria.

Inclusion Criteria	Exclusion Criteria
Individuals (≥40 years) with major depressive episodes according to the diagnostic criteria of Diagnostic and Statistical Manual of Mental Disorders IV-TR (before 2013) or Diagnostic and Statistical Manual of Mental Disorders 5 (after 2013)	Psychiatric disorders other than major depressive disease and/or substance use disorder (current or past)
No previous cardiovascular diseases (coronary heart disease, heart failure, cerebrovascular disease, and peripheral vascular disease)	Acute and/or uncontrolled somatic, infectious, or inflammatory pathologies
No pregnancy	Brain lesions induced by neurological disorders and/or trauma
No parasomnias, central disorders of hypersomnolence, or obstructive sleep apnea syndrome already treated in the past or being treated before admission to the Sleep Unit and sleep apnea syndrome with predominantly central component	Craniofacial and/or thoracic malformations

**Table 2:** Polysomnographic parameters (n = 607).

	Whole Sample (n = 607)	MDD with Low Cardiovascular Risk (n = 362)	MDD with High Cardiovascular Risk (n = 245)	* p * -Value
Sleep latency (min)	34.7 (18.3–63.5)	31.3 (18.0–59.0)	38.0 (19.0–68.0)	0.056
Sleep efficiency (%)	77.2 (69.1–84.4)	78.8 (70.9–85.4)	75.1 (65.1–81.8)	<0.001
Sleep period time (min)	445.5 (410.5–482.0)	449.8 (420.5–486.5)	441.0 (394.3–478.0)	0.001
Total sleep time (min)	384.7 (336.3–423.5)	392.0 (350.5–429.0)	374.0 (323.7–414.5)	<0.001
% stage 1	7.6 (5.2–10.9)	7.4 (5.1–10.2)	8.4 (5.4–12.0)	0.021
% stage 2	54.7 (47.7–62.3)	54.1 (47.6–61.8)	55.7 (48.2–62.5)	0.532
% stage 3	3.3 (0.2–10.0)	5.1 (0.8–11.3)	1.2 (0.0–6.9)	<0.001
% rapid eye movement sleep	16.0 (11.3–20.0)	16.5 (12.3–20.3)	14.7 (9.9–19.9)	0.006
Latency of rapid eye movement sleep (min)	92.5 (64.5–162.0)	92.5 (64.4–149.6)	92.5 (65.0–176.0)	0.700
% wake after sleep onset	12.1 (7.1–19.4)	11.3 (6.5–17.7)	13.4 (8.4–21.4)	0.001
Number of awakenings	30 (21–45)	29 (21–43)	32 (21–47)	0.077
Micro-arousal index	10 (6–16)	9 (6–13)	12 (8–22)	<0.001
Apnea–hypopnea index	3 (1–11)	2 (1–6)	7 (3–21)	<0.001
Oxygen desaturation index	2 (0–5)	1 (0–3)	4 (1–9)	<0.001
Total time under 90% of SaO_2_ (min)	1.5 (0.0–19.0)	0.5 (0.0–6.5)	10.0 (1.0–48.7)	<0.001
PLMs index	2 (0–11)	2 (0–9)	3 (0–14)	0.270
	Median (P25–P75)	Median (P25–P75)	Median (P25–P75)	Wilcoxon Test

MDD = major depressive disease, PLMs = periodic limb movements during sleep.

**Table 3:** Sample description (n = 607).

Variables	Categories	%	MDD with Low Cardiovascular Risk	MDD with High Cardiovascular Risk	* p * -Value Chi Square Tests	OR (CI 95%)	* p * -Value
Gender	Female (n = 320)	52.70%	69.60%	27.80%	<0.001	1	<0.001
	Male (n = 287)	47.30%	30.40%	72.20%		5.96 (4.17 to 8.53)	
Body mass index (kg/m^2^)	<25 (n = 177)	29.20%	38.10%	15.90%	<0.001	1	<0.001
	≥25 & <30 (n = 205)	33.80%	32.30%	35.90%		2.66 (1.70 to 4.18)	
	≥30 (n = 225)	37.00%	29.60%	48.20%		3.90 (2.51 to 6.07)	
Age (years)	<60 (n = 507)	83.50%	91.40%	71.80%	<0.001	1	<0.001
	≥60 (n = 100)	16.50%	8.60%	28.20%		4.19 (2.64 to 6.64)	
Antidepressant therapy	No (n = 312)	51.40%	51.40%	51.40%	0.991	1	0.991
	Yes (n = 295)	48.60%	48.60%	48.60%		1.00 (0.72 to 1.38)	
Benzodiazepine receptor agonists	No (n = 448)	73.80%	72.60%	75.50%	0.432	1	0.432
	Yes (n = 159)	26.20%	27.40%	24.50%		0.86 (0.59 to 1.25)	
Smoking	No (n = 476)	78.40%	83.40%	71.00%	<0.001	1	<0.001
	Yes (n = 131)	21.60%	16.60%	29.00%		2.05 (1.39 to 3.04)	
Alcohol	No (n = 415)	68.40%	71.50%	63.70%	0.041	1	0.041
	Yes (n = 192)	31.60%	28.50%	36.30%		1.43 (1.01 to 2.03)	
COMISA status	No insomnia and/or OSAS (n = 94)	15.50%	18.20%	11.40%	<0.001	1	<0.001
	Insomnia alone (n = 258)	42.50%	52.50%	27.80%		0.84 (0.50 to 1.42)	
	OSAS alone (n = 106)	17.50%	10.50%	27.80%		4.22 (2.33 to 7.64)	
	COMISA (n = 149)	24.50%	18.80%	33.00%		2.81 (1.62 to 4.85)	
Sleep movement disorders	No (n = 466)	76.80%	79.80%	72.40%	0.002	1	0.03
	Moderate to severe PLMs alone (n = 44)	7.20%	7.50%	6.90%		1.03 (0.54 to 1.94)	
	RLS alone or combined with PLMs (n = 97)	16.00%	12.70%	20.80%		1.81 (1.17 to 2.81)	
Excessive daytime sleepiness	No (n = 289)	47.60%	47.20%	48.20%	0.823	1	0.823
	Yes (n = 318)	52.40%	52.80%	51.80%		0.96 (0.70 to 1.33)	
Type 2 diabetes	No (n = 511)	84.20%	95.60%	67.40%	<0.001	1	<0.001
	Yes (n = 96)	15.80%	4.40%	32.60%		10.48 (5.94 to 18.50)	
Hypertension status	No (n = 300)	49.40%	67.40%	22.80%	<0.001	1	<0.001
	Untreated (n = 104)	17.10%	13.00%	23.30%		5.28 (3.26 to 8.57)	
	Controlled (n = 125)	20.60%	15.20%	28.60%		5.55 (3.51 to 8.76)	
	Uncontrolled (n = 78)	12.90%	4.40%	25.30%		16.88 (9.07 to 31.44)	
Dyslipidemia status	No (n = 296)	48.80%	62.40%	28.60%	<0.001	1	<0.001
	Without statin therapy (n = 216)	35.60%	28.50%	46.10%		3.54 (2.43 to 5.17)	
	With statin therapy (n = 95)	15.60%	9.10%	25.30%		6.07 (3.68 to 10.00)	
Cardiovascular comorbidities	No (n = 549)	90.40%	93.10%	86.50%	0.007	1	0.008
	Yes (n = 58)	9.60%	6.90%	13.50%		2.10 (1.21 to 3.63)	
Aspirin therapy	No (n = 545)	89.80%	94.70%	82.50%	<0.001	1	<0.001
	Yes (n = 62)	10.20%	5.30%	17.50%		3.84 (2.18 to 6.78)	
CRP (mg/L)	<1 (n = 153)	25.20%	30.10%	18.00%	0.001	1	0.001
	≥ 1 (n = 454)	74.80%	69.90%	82.00%		1.97 (1.32 to 2.92)	
Depression severity	Mild to moderate (n = 432)	71.20%	68.80%	74.70%	0.115	1	0.115
	Severe (n = 175)	28.80%	31.20%	25.30%		0.75 (0.52 to 1.07)	
Cardiovascular risk	Low (n = 362)	59.60%					
	High (n = 245)	40.40%					
	Median				Wilcoxon test		
	(P25–P75)						
BMI (kg/m^2^)	27.8 (24.4–32.1)		26.4 (23.2–30.8)	29.7 (26.8–33.5)	<0.001		
Age (years)	50 (45–56)		48 (43–53)	55 (49–60)	<0.001		
ESS	11 (7–14)		11 (6–15)	11 (8–14)	0.692		
BDI	12 (10–16)		13 (10–17)	11 (9–16)	0.005		
ISI	18 (14–21)		19 (15–22)	17 (14–20)	<0.001		
CRP (mg/L)	1.8 (1.0–3.6)		1.6 (0.8–3.5)	2.0 (1.1–3.8)	0.002		
Framingham Risk Score (%)	7.9 (4.3–14.1)		4.9 (3.1–7.1)	16.4 (12.6–23.0)	<0.001		

MDD = major depressive disease, OSAS = obstructive sleep apnea syndrome, COMISA = comorbid insomnia and sleep apnea, CRP = C-Reactive Protein, PLMs = periodic limb movements during sleep, RLS = restless legs syndrome, ESS = Epworth sleepiness scale, BDI = Beck Depression Inventory, ISI = Insomnia Severity Index.

**Table 4:** Multivariate analyzes (n = 607).

Variables	Model 1 OR Adjusted (CI 95%)	*p*-Value	Model 2 OR Adjusted (CI 95%)	*p*-Value	Model 3 OR Adjusted (CI 95%)	*p*-Value	Model 4 OR Adjusted (CI 95%)	*p*-Value
COMISA status		0.006		0.017		0.015		0.013
No insomnia and/or OSAS	1		1		1		1	
Insomnia alone	1.20 (0.64 to 2.24)		1.06 (0.49 to 2.32)		1.08 (0.49 to 2.37)		1.08 (0.49 to 2.37)	
OSAS alone	2.23 (1.07 to 4.62)		2.29 (0.91 to 5.74)		2.36 (0.93 to 5.95)		2.40 (0.95 to 6.08)	
COMISA	2.53 (1.30 to 4.93)		2.61 (1.15 to 5.95)		2.67 (1.17 to 6.13)		2.70 (1.18 to 6.19)	

Model 1 = model adjusted for male sex, overweight, obesity, age ≥ 60 years, smoking, and alcohol consumption. Model 2 = model adjusted for male sex, overweight, obesity, age ≥ 60 years, smoking, alcohol consumption, type 2 diabetes, untreated hypertension, controlled hypertension, uncontrolled hypertension, dyslipidemia without statin therapy, dyslipidemia with dyslipidemia, cardiovascular comorbidities, and aspirin therapy. Model 3 = model adjusted for male sex, overweight, obesity, age ≥ 60 years, smoking, alcohol consumption, type 2 diabetes, untreated hypertension, controlled hypertension, uncontrolled hypertension, dyslipidemia without statin therapy, dyslipidemia with dyslipidemia, cardiovascular comorbidities, aspirin therapy, and CRP levels ≥ 1 mg/L. Model 4 = model adjusted for male sex, overweight, obesity, age ≥ 60 years, smoking, alcohol consumption, type 2 diabetes, untreated hypertension, controlled hypertension, uncontrolled hypertension, dyslipidemia without statin therapy, dyslipidemia with dyslipidemia, cardiovascular comorbidities, aspirin therapy, CRP levels ≥ 1 mg/L, and RLS alone or combined with periodic limb movement index ≥15/h. COMISA = comorbid insomnia and sleep apnea, CRP = C-Reactive Protein, OSAS = obstructive sleep apnea syndrome, RLS = restless legs syndrome.

**Table 5:** Summary of the comparison of the main results of this study with the literature.

Results of This Study	Comparison with Literature	Explanations
A total of 40.4% of MDD patients presented high 10-year cardiovascular risk.	A total of 8.3% of individuals from the general population presented high 10-year cardiovascular risk [39].	MDD patients are a subpopulation at high cardiovascular risk [3]
	A total of 31.9% of patients with mood disorders from the study by Yakar et al. (2018) and 23.4% of patients with mood disorders from the study by Garcia-Portilla et al. (2009) presented high 10-year cardiovascular risk [40,41].	Recruitment of patients characterized by a less severe cardiovascular profile in the studies by Yakar et al. (2018) and Garcia-Portilla et al. (2009) [40,41]
	A total of 59.3% of patients with mood disorders from the study by Slomka et al. (2012) presented high 10-year cardiovascular risk [42].	Recruitment of patients characterized by a more severe cardiovascular profile in the study by Slomka et al. (2012) [42]
A total of 58.4% of MDD patients with OSAS presented a comorbid ID.	A total of 41.1% of diabetic patients with OSAS from the study by Hein et al. (2022), 38.4% of hypertensive patients with OSAS from the study by Draelants et al. (2023), and 40.0% of apneic patients from the study by Hein et al. (2022) presented a comorbid ID [12,13,18].	Existence of pathophysiological mechanisms specific to MDD (vulnerability to stress-related disturbances and some neurobiological factors) favoring the more frequent occurrence of comorbid ID [46,47,48]
For MDD patients, high 10-year cardiovascular risk was significantly associated with COMISA but was not significantly associated with ID or OSAS alone.	Results similar to those of the studies by Hein et al. (2022) for diabetic patients, by Draelants et al. (2023) for hypertensive patients, and Hein et al. (2022) for apneic patients [12,13,18].	Existence of a negative synergistic action between OSAS and ID on cardiovascular outcome [50,51,52]

ID = insomnia disorder, MDD = major depressive disease, OSAS = obstructive sleep apnea syndrome.

## Data Availability

The corresponding author may provide the data for this study upon reasonable request.

## Supplementary Materials

The following supporting information can be downloaded at: https://www.mdpi.com/article/10.3390/life14050644/s1, S1: Ambulatory care journeys of MDD patients; S2: Description of traditional cardiovascular risk factors systematically screened during stays in the Sleep Unit; S3: Description of the standardized questionnaire series; S4: Description of the stay in the Sleep Unit and the equipment used for the polysomnographic recording; S5: Description of the scoring criteria used by the specialized technicians of the Sleep Unit; S6: Diagnostic criteria used for insomnia disorder; S7: Description of the confounders used during the different statistical analyzes. References [66,67,68,69,70,71,72,73,74,75] are cited in the supplementary materials.
