# Supplementary material for: Cardiovascular Outcome in Patients with Major Depression: Role of Obstructive Sleep Apnea Syndrome, Insomnia Disorder, and COMISA
Source: Life (Basel). 2024 May 19;14(5):644. doi: 10.3390/life14050644 (PMC11123427; doi:10.3390/life14050644)
Supplement: Supplementary file 1 [file life-14-00644-s001.zip › life-2989998-supplementary.pdf]

Supplementary Materials

S 1

Ambulatory care journeys of MDD patients

All major depressed patients recruited for this study were initially referred to the Sleep Laboratory by physicians specialized in sleep medicine after an outpatient consultation during which a preliminary assessment of their complaints related to sleep, their ongoing psychotropic/somatic treatments and their somatic/psychiatric comorbidities was systematically performed to allow a first diagnostic hypothesis. Following this initial assessment, a polysomnographic recording was programmed in all these major depressed patients to allow an objective assessment of their sleep complaints and to exclude the presence of comorbid sleep disorders that could negatively impact mood regulation.

S 2

Description of traditional cardiovascular risk factors systematically screened during stays in the Sleep Unit

In all major depressed patients included in this study, conventional cardiovascular risk factors were considered present according to the following criteria:

- Type 2 diabetes (*American Diabetes Association* diagnostic criteria): glycated hemoglobin (HbA1c) ≥6.5% or fasting plasma glucose ≥126 mg/dl or two-hour plasma glucose ≥200 mg/dl during an oral glucose tolerance test or random plasma glucose ≥200 mg/dl in patients with classic symptoms of hyperglycemia or self-reported diagnosis of clinically demonstrated type 2 diabetes or taking antidiabetic medication [66]. In the absence of unequivocal hyperglycemia, criteria 1–3 should be confirmed by repeat testing. In addition, diabetes must have begun in adulthood [66].
- Hypertension (*World Health Organization* diagnostic criteria): mean systolic blood pressure ≥140 mmHg or mean diastolic blood pressure ≥90 mmHg or self-reported diagnosis of clinically demonstrated hypertension or use of antihypertensive medication [67]. In treated hypertensive individuals, controlled hypertension was defined as the presence of mean systolic blood pressure <140 mmHg and mean diastolic blood pressure <90 mmHg whereas uncontrolled hypertension was defined as the presence of mean systolic blood pressure ≥140 mmHg and/or mean diastolic blood pressure ≥90 mmHg [67].
- Dyslipidemia (*International Diabetes Federation* diagnostic criteria): plasma triglyceride level ≥150 mg/dl or plasma HDL-cholesterol level <40 mg/dl for men

or plasma HDL-cholesterol level <50 mg/dl for women or use of treatment for dyslipidemia [68].

- Cardiovascular comorbidities (excluding hypertension): presence of one or more comorbid cardiovascular pathologies (cardiac arrhythmias, non-ischemic cardiomyopathy, cardiac valve disease and history of cardiac surgery).

S 3

Description of the standardized questionnaire series

- The Beck Depression Inventory (reduced to 13 items) was used to investigate the presence of depressive symptoms. The 13 items of this scale may be scored from 0 to 3, which mean that the total score may vary from 0 to 39. A final score of 0-4 indicates an absence of depressive symptoms, 5-7 mild depressive symptoms, 8-15 moderate depressive symptoms, and ≥16 severe depressive symptoms [27].
- The Insomnia Severity Index was used to investigate the severity of insomnia complaints. The 7 items of this index may be scored from 0 to 4, which mean that the total score may vary from 0 to 28. A final score of 0-7 indicates an absence of insomnia complaints, 8-14 subclinical insomnia complaints, 15-21 moderate insomnia complaints, and 22-28 severe insomnia complaints [28].
- The Epworth Sleepiness Scale was used to investigate daytime sleepiness. The 8 items of this scale assessing sleepiness in different daytime situations may be scored from 0 to 3, which mean that the total score may vary from 0 to 24. A final score greater than 10 indicates excessive daytime sleepiness [29].

S 4

Description of the stay in the Sleep Unit and the equipment used for the polysomnographic recording

1/Stay conditions at the Sleep Laboratory

The patients went to bed between 22:00 - 24:00 and got up between 6:00 - 8:00, following their usual schedule. During bedtime hours, the subjects were recumbent and the lights were turned off. Daytime naps were not permitted.

2/Applied polysomnography-montage

- Two electro-oculogram channels
- Three electroencephalogram channels
- One submental electromyogram channel
- Electrocardiogram
- Pressure cannula to detect the oro-nasal airflow
- Finger pulse-oximetry
- Microphone to record breathing sounds and snoring
- Plethysmographic inductive belts to measure thoracic and abdominal breathing
- Anterior tibialis electrodes

S 5

Description of the scoring criteria used by the specialized technicians of the Sleep Unit

Obstructive apneas were scored if the decrease in air flow was ≥90% for at least 10 seconds whereas obstructive hypopneas were scored if the decrease in airflow was ≥30% for at least 10 seconds with a decrease in oxygen saturation of 3% or followed by micro-arousal [32]. The obstructive apnea-hypopnea index corresponds to the total number of obstructive apneas and hypopneas divided by the period of sleep in hours [32].

Periodic limb movements were scored on the basis of the following strict criteria: 1) duration between 0.5 to 10 seconds, 2) interval between 5 and 90 seconds from leg movement onset and 3) movements had to be part of a series of ≥4 consecutive movements meeting these criteria. According to scoring rules, limb movements associated with respiratory events should not be considered as periodic limb movements and cannot be included in the calculation of the periodic limb movement index [33]. Periodic limb movement index corresponds to the total number of periodic limb movements divided by period of sleep in hours [33].

S 6

Diagnostic criteria used for insomnia disorder [37]

Criteria	
A	The individual reports one or more of the following sleep related complaints:<br>1. difficulty initiating sleep<br>2. difficulty maintaining sleep<br>3. waking up too early<br>4. sleep that is chronically nonrestorative or poor in quality
B	The above sleep difficulty occurs despite adequate opportunity and circumstances for sleep
C	At least one of the following forms of daytime impairment related to the nighttime sleep difficulty is reported by the individual:<br>1. fatigue/dizziness<br>2. attention, concentration or memory impairment<br>3. social/vocational dysfunction or poor school performance<br>4. mood disturbance/irritability<br>5. daytime sleepiness<br>6. motivation/energy/initiative reduction<br>7. proneness for errors/accidents at work or while driving<br>8. tension headaches and/or GI symptoms in response to sleep loss<br>9. concerns or worries about sleep

S 7

Description of the confounders used during the different statistical analyzes

After a review of the literature on cardiovascular risk factors in major depressed patients and in the general population [12,13,18,65,69-75], potential confounders included in this study were body mass index categories (categorized: <25 kg/m^2, ≥25 & <30 kg/m^2, ≥30 kg/m^2), age (categorized: <60 years, ≥60 years), sleep movement disorders (categorized: absent, periodic limb movement syndrome alone, restless legs syndrome alone or combined with periodic limb movement index ≥15/hour), hypertension status (categorized: absent, untreated, controlled, uncontrolled), dyslipidemia status (categorized: absent, without statin therapy, with statin therapy), CRP levels (categorized: <1 mg/L, ≥1 mg/L), depression severity (categorized: mild to moderate, severe) and as binary variables: gender, antidepressant therapy, benzodiazepine receptor agonists, alcohol consumption, smoking, excessive daytime sleepiness, type 2 diabetes, cardiovascular comorbidities and aspirin therapy.
